# Predicting the Extension of Biomedical Ontologies

**DOI:** 10.1371/journal.pcbi.1002630

**Published:** 2012-09-13

**Authors:** Catia Pesquita, Francisco M. Couto

**Affiliations:** Faculty of Sciences, University of Lisboa, Lisboa, Portugal; University of Colorado School of Medicine, United States of America

## Abstract

Developing and extending a biomedical ontology is a very demanding task that can never be considered complete given our ever-evolving understanding of the life sciences. Extension in particular can benefit from the automation of some of its steps, thus releasing experts to focus on harder tasks. Here we present a strategy to support the automation of change capturing within ontology extension where the need for new concepts or relations is identified. Our strategy is based on predicting areas of an ontology that will undergo extension in a future version by applying supervised learning over features of previous ontology versions. We used the Gene Ontology as our test bed and obtained encouraging results with average f-measure reaching 0.79 for a subset of biological process terms. Our strategy was also able to outperform state of the art change capturing methods. In addition we have identified several issues concerning prediction of ontology evolution, and have delineated a general framework for ontology extension prediction. Our strategy can be applied to any biomedical ontology with versioning, to help focus either manual or semi-automated extension methods on areas of the ontology that need extension.

## Author Summary

Biomedical knowledge is complex and in constant evolution and growth, making it difficult for researchers to keep up with novel discoveries. Ontologies have become essential to help with this issue since they provide a standardized format to describe knowledge that facilitates its storing, sharing and computational analysis. However, the effort to keep a biomedical ontology up-to-date is a demanding and costly task involving several experts. Much of this effort is dedicated to the addition of new elements to extend the ontology to cover new areas of knowledge. We have developed an automated methodology to identify areas of the ontology that need extension based on past versions of the ontology as well as external data such as references in scientific literature and ontology usage. This can be a valuable help to semi-automated ontology extension systems, since they can focus on the subdomains of the identified ontology areas thus reducing the amount of information to process, which in turn releases ontology developers to focus on more complex ontology evolution tasks. By contributing to a faster rate of ontology evolution, we hope to positively impact ontology-based applications such as natural language processing, computer reasoning, information integration or semantic querying of heterogenous data.

## Introduction

Despite the last decade's efforts to structure and organize the deluge of biomedical data brought on by high throughput techniques, there are still many issues that challenge biomedical knowledge discovery and management [1].

On one hand, most scientific knowledge is still present only in natural language text in the form of scientific publications, whose number grows nearly exponentially making it necessary to employ text mining techniques if we are ever to aspire at keeping up. However, the natural ambiguity and subjectivity of natural language hinders the automated processing of scientific publications. On the other hand, although there is a large number of databases to store biomedical data, the effort to achieve interoperability between them is still lagging behind, given that most resources, particularly the older ones, were developed in a completely independent fashion, and the efforts to connect them to other resources are still insufficient.

One very important breakthrough for both areas, was the development of biomedical ontologies (bio-ontologies). They support both issues, by providing unequivocal and structured models of specific domains, which is fundamental to resolve semantic ambiguities in text mining and also to serve as a common background to biomedical databases.

The development of a biomedical ontology, or other domain ontologies, is a very demanding process that requires both expertise in the domain to model, as well as in ontology design. This means that people from different backgrounds, such as biology, philosophy and computer science should be involved in the process of creating an ontology. However, specific biomedical ontologies are usually built by small teams of life sciences researchers, with little experience in ontology design. They are responsible for first, agreeing on the precise limits of the domain to model; second, defining the structure and complexity of the model; and finally, building the ontology itself by creating the concepts, relations and other axioms it might contain [2].

Several methodologies have been developed to help build ontologies [2]–[5], with the most well-known ontology editors in the biomedical ontologies community being Protégé [6] and OBO-Edit [7]. Nevertheless, ontology development remains a mostly manual and labor-intensive task, which is magnified if the domain to model is as dynamic and complex as the life sciences. Biomedical ontologies can never be considered complete, always having to adapt to our new understanding of biological knowledge. This forces biomedical ontology development to be an iterative process [8], [9] in order to keep up with the dynamic and evolving domain. In fact, one of the tenets of the Open Biological and Biomedical Ontologies (OBO) Foundry, an initiative that establishes a set of principles for ontology development in the biomedical domain, is that an ontology should be maintained in light of scientific advance [10].

This ontology evolution [11] is a continuous effort, requiring large investments of both time and resources with each new version that is produced. Moreover, many biomedical ontologies cover large and complex domains which magnifies the effort required, even when considering highly successful ontologies, such as the Gene Ontology [12], where a large community is engaged in its creation. These challenges create the need for semi-automated systems that are able to support ontology engineers in the task of ontology evolution. However, a significant majority of efforts in this area is not concerned with evolving an existing ontology, but rather in learning a new ontology from scratch, usually from textual resources [13]–[19]. Nevertheless, they can in principle be used for ontology extension as well. These approaches usually depend on either a manually selected corpus of texts to be used as input to narrow down the domain of interest, or process large corpus with generic domains.

A relevant process of ontology evolution is the addition of new elements, i.e. ontology extension. Ontology extension is particularly relevant in fast growing domains such as biomedicine, where new knowledge is created everyday. The first step in this is to identify the changes that need to be performed: change capturing. This is vitally different from a general ontology learning process that handles the whole domain at once, in that it is focused on specific areas within the domain of the ontology to be extended.

In this paper we present a methodology that addresses change capturing by predicting ontology extension. The fact that these changes can in principle be semi-automatically discovered by analyzing the ontology data and its usage motivated the present work. It is a supervised learning based strategy that predicts the areas of the ontology that will undergo extension in a future version, based on previous versions of the ontology. By pinpointing which areas of the ontology are more likely to undergo extension, this methodology can be integrated into ontology extension approaches, both manual and semi-automated, to provide a focus for extension efforts and thus contributing to ease the burden of keeping an ontology up-to-date.

The primary goal of our methodology is to function as a first step in automated ontology learning or extension systems. Ontology learning systems, usually rely on the analysis of a manually constructed corpus of documents pertaining to the domain of interest and their performance is closely coupled to the relevance of these documents. The challenge of focusing the ontology given an heterogenous corpus in ontology learning has been identified [20], a challenge that is amplified when it comes to ontology extension of large ontologies, as is the case of many biomedical ontologies. A comprehensive corpus for these ontologies would be quite large and building and then processing it would be cumbersome. By applying our strategy, ontology developers can identify subdomains to extend, create tailored corpus for them, and then run the learning systems over them, reducing the amount of data they have to process to identify new concepts. Another option for ontology extension is based on ontology matching, which can be used to support the integration of elements from other ontologies. Our strategy can also be interesting in this case, since by pinpointing the areas to extend, it can help to narrow down on specific ontologies to match.

Our main contribution for ontology developers lies in the speeding of the process of extension in these areas, thus releasing the experts to focus on more complex ontology evolution issues. We have chosen to evaluate our approach using the Gene Ontology, since it provides many versions spanning a number of years, and is perhaps one of the best known and widely used biomedical ontologies.

In the remainder of this section we will introduce some basic concepts, present related work and describe the Gene Ontology.

### Ontology Evolution and Extension

Ontology evolution can be defined as the process of modifying an ontology in response to a certain change in the domain or its conceptualization [21]. These include (1) changes in the portion of the real world they model, (2) a reassessment of the relevance of some element to the ontology, (3) the uncovering of information previously unavailable, or (4) a need to correct previous mistakes [22]. In general, the evolution of biomedical ontologies is mainly concerned with the third and fourth types, given the dynamic nature of biological knowledge production, everyday new discoveries are published, rendering some facts obsolete and bringing new knowledge to light.

Ontology evolution comprises several different processes, based on the type of change transformations they employ over ontology elements: add, remove or modify. While adding new elements is mostly employed in response to a change of the first or third type, removing elements is often related to the first, second and fourth types. Modifying existing elements can belong to any of the four kinds and ultimately be seen as a compound change of removing one element and adding a slightly different new one. In this work we are only concerned with change transformations that add new elements to the ontology, thereby extending it.

Although [21] and [23] provide an exhaustive terminology for ontology change, some finer grained aspects of ontology evolution remained confusing, with several terms being used in an ambiguous fashion. In a previous work [24] we defined and distinguished three terms related to ontology changes concerned with the addition of new elements: *ontology extension*, *ontology refinement* and *ontology enrichment*. Although ontology extension is often used interchangeably with both refinement and enrichment, we defined them as follows:

**Ontology extension** is the process by which new single elements are added to an existing ontology.

Thus, ontology extension is concerned with elementary changes of the addition type. Many reasons can motivate such a change, such as new discoveries, access to previously unavailable information sources, a change in the viewpoint or usage of the ontology, a change in the level of refinement of the ontology, etc, but they all rely on the finding of new knowledge. Ontology extension encompasses both ontology refinement and ontology enrichment.

**Ontology refinement** is the addition of new concepts to an ontology, where a new subsumption relation is established between an existing concept and the new concept. For instance, the addition of the concept “mitochondrial fusion" as a subconcept of “organelle fusion" in the biological process branch of the Gene Ontology.

**Ontology enrichment** is the process by which non-taxonomical relations or other axioms are added to an existing ontology. For instance, the addition of the relation “regulates" between the GO concepts “regulation of mitochondrial translation" and “mitochondrial translation".

### Change Capturing

Before these changes are actually performed, the need for the change must be identified. This is the first step of any ontology evolution process, the change capturing phase, and it can be based on explicit or implicit requirements [15]. Explicit requirements correspond to those made by the ontology developers or to requests made by end-users. Implicit requirements correspond to those that can be uncovered by change discovery. Stojanovic [25] lists a series of guidelines for change capturing, organized into three types according to the kind of data they exploit, to which Castaño et al. [26] add a fourth:

**structure-driven:** which are derived from the structure of the ontology, e.g. ‘A class with a single subclass should be merged with its subclass’.

**data-driven:** which correspond to implicit changes in the domain and are discovered through the analysis of the instances belonging to the ontology, e.g. ‘A class with many instances is a candidate for being split into subclasses and its instances distributed among newly generated classes’.

**usage-driven:** which are deduced from the usage patterns of the ontology in the knowledge management system e.g. classes that have not been retrieved in a long time might be out of date.

**discovery-driven:** which is applied when a new instance cannot be described by the ontology classes, and new classes are identified using external resources.

### Related Work

Although there is a large body of work on ontology evolution (for a review see [27]), there are few works on the change capturing phase. Stojanovic at al. [28] proposed an approach to ontology evolution that is based on optimizing the ontology according to the end-users needs. They track end-users interactions with an ontology-based application to collect useful information that can be used to assess what the main interests of the end-users are. Their approach is then a usage-driven change discovery, which focuses on discovering anomalies in the design of an ontology, whose repairing improves the usability of this ontology. This uses several measures, based on querying and browsing of an ontology-based application.

Browsing-based measures are based on the user's browsing of links between ontology concepts. They define the usage of two concepts 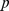 and 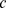 as the number of times the link between them has been browsed, where the 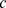 is a subconcept of a concept 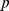. This concept is used in four measures for estimating the uniformity (balance) of the usage of a link regarding the link neighborhood: (1) SiblingUniformity represents the ratio between the usage of a link and the usage of all links, which have the common source node with that link (the so-called sibling links); (2) ChildrenUniformity stands for the ratio between the sum of the usage of all the links whose source node is the given node and the sum of the usage of a node through all incoming links into this node. (3) ParentUniformity is the ratio between the usage of a link and the usage of all links which have the common destination node with that link, and (4) UpDownUniformity characterizes the ratio between the usage of a link in two opposite directions, i.e. in browsing down and browsing up through a hierarchy.

Another usage-driven strategy was proposed by [29] in the context of the evolution of multiple personal ontologies, which is based on a user's ratings of concepts and axioms.

Also relevant for our work is the investigation of ontology evolution in biomedical ontologies.

In [30], the author applied a previously proposed strategy, Evolutionary Terminology Auditing (ETA) [22] to assess the quality of GO using reality as benchmark. This strategy can be used not so much to demonstrate how good an individual version of a terminology is, but rather to measure how much it has been improved (or believed to have been improved) as compared to its predecessor. This is based on matches and mismatches between ontology versions, and their motivations, which are expressed by 17 possible configurations split into four groups, denoting, respectively, the presence or absence of a term and whether the presence or absence of a term in a terminology is justified or unjustified. Of these 17 configurations only two correspond to a need for extension, in which an entity is missing and it is real and relevant for the ontology.

[31] proposes an approach to automatically discover evolving or stable regions of ontologies. This approach is based on a cost model for changes between ontology versions and is able to identify regions that have been undergoing (or not) extensive changes.

On a previous study we delineated a framework to analyze ontology extension and used it as a background for investigating the feasibility of predicting ontology extension based on a set of rules [24]. In predicting ontology evolution we were aiming at developing a methodology for change capturing. We based our set of rules on the guidelines proposed by [25] following [8] for ontology development, namely:

A concept with many instances is a candidate for being split into subconcepts and its instances distributed among newly generated concepts.

If a class has only one direct subclass there may be a modeling problem or the ontology is not complete.

If there are more than a dozen subclasses for a given class then additional intermediate categories may be necessary.

Based on these we created a set of rules for predicting the extension of the Gene Ontology:

**Rule 1:** A class with less subclasses than its siblings is a candidate for refinement

**Rule 2:** A class with more total annotations than its siblings is a candidate for refinement

**Rule 3:** A class with more manual annotations than its siblings is a candidate for refinement

Application of these rules to several versions of the Gene Ontology yielded very poor prediction results, highlighting the need for more complex approaches to model this issue.

The strategy we present here is unlike previously described works, since we use metrics of previous ontology versions to support prediction, whereas change capturing approaches are based on manually derived rules and ontology evolution approaches analyze evolution of existing ontology versions.

### Gene Ontology

The Gene Ontology (GO) is currently the most successful case of ontology application in bioinformatics and provides a controlled vocabulary to describe functional aspects of gene products under three distinct ontologies: biological process, molecular function and cellular component. GO terms are structured in a directed acyclic graph with its hierarchical backbone being composed of 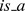 and 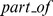 relations.

GO is used to annotated gene products, and these annotations are compiled by the Gene Ontology Annotation project (GOA). GO annotations are assigned an evidence code which identifies the kind of evidence supporting the annotation. Although over a dozen evidence codes exist, the most relevant distinction between them is whether they are manually assigned by a curator or inferred electronically. Electronic annotations are generally considered to be of lower quality than manual ones, but compose the vast majority of present GO annotations (over 97%). Another relevant aspect of annotations is whether they can be considered direct, i.e. the annotation was made precisely to that GO term; or indirect, i.e. the annotation was made to a subconcept of that GO term, from which we can deduce that there is also an annotation to all of its superconcepts.

GO also provides a cut-down version of the GO ontologies, GO Slims, which contain a subset of the terms in the whole GO to give a broad overview of the ontology content without the detail of the specific fine grained terms.

There are about one hundred contributors to GO between the GO Consortium and GO Associates, and they are expected to contribute regularly towards the content of GO. Other GO users can also contribute by suggesting new terms via Sourceforge.net, however the majority of content requests are made by GO team members [32]. GO team experts base their decision to change the ontology on the following precepts:

- working closely with the reference genome annotation group to ensure that areas that are known to undergo intense annotation in the near future are updated
- listening to the biological community
- ensuring that emerging genomes have the necessary classes to support their needs

Although some steps have been taken in the direction of automatizing some aspects of GO evolution, namely the extension of GO with computable logical definitions including cross-references to other ontologies [33] and a new method to optimize the distribution of the information within the GO structure [34], the evolution of GO remains challenging given the complex decision-making processes involved [35].

## Methods

### Data

Following our previous work [24], we used 15 versions of the Gene Ontology spanning a period of seven years. Table 1 identifies these versions, and describes a few general statistics about them. The versions have a six-month interval between them or as close to that as possible, since not all versions have a full database available from the Gene Ontology archive.

**Table 1:** Description of Gene Ontology versions.

ontology version	n. terms	n. relations	max depth	avg depth	deletions*	insertions*	total annotations	manual annotations
Jan 2005	17K	26K	17	6.8	N/A	N/A	6.0 M	0.50 M
Jul 2005	18K	28K	19	7.0	111	885	7.1 M	0.62 M
Jan 2006	19K	30K	18	7.0	42	1311	7.3 M	0.56 M
Jul 2006	20K	31K	18	7.0	20	578	9.0 M	0.56 M
Jan 2007	22K	35K	18	7.2	97	2079	10.4 M	0.62 M
Jun 2007	23K	38K	18	6.9	131	1454	12.4 M	0.66 M
Jan 2008	24K	40K	18	4.9	153	1674	19.0 M	0.73 M
Jul 2008	25K	44K	18	4.9	104	807	23.0 M	0.78 M
Jan 2009	27K	47K	18	4.9	17	1415	24.7 M	0.79 M
Aug 2009	28K	51K	18	5.0	77	1487	33.0 M	0.87 M
Jan 2010	29K	54K	19	4.9	61	1476	33.5 M	0.91 M
Jul 2010	32K	57K	15	3.9	31	1302	60.5 M	1.06 M
Jan 2011	33K	60K	15	4.01	106	2698	54.4 M	1.23 M
Jul 2011	34K	63K	15	4.03	48	1208	63.8 M	1.35 M
Jan 2012	36K	65K	15	4.05	32	1113	77.8 M	1.41 M

* with respect to the version in the line above.

### Extension Prediction Strategy

The intuition behind our proposed strategy is that information encoded in the ontology or its annotation resources can be used to support the prediction of ontology areas that will be extended in a future version. This notion is inspired by change capturing strategies that are based on implicit requirements. However in the existing change capturing approaches, these requirements are manually defined based on expert knowledge. Our system attempts to go beyond this, by trying to learn these requirements based on previous extension events using supervised learning.

In our test case using GO, we use as attributes for learning a series of ontology features based on structural, annotation or citation data. These are calculated for each GO term and then used to train a model able to capture whether a term would be extended in a following version of GO.

Structural features give information on the position of a term and the surrounding structure of the ontology, such as height (i.e. distance to a leaf term), number of sibling or children terms. A term is considered to be direct child if it is connected to its parent by an *is_a* or *part_of* relation, but the total of children of a term encompasses all descendants regardless of the number of links between them. Annotation features are based on the number of annotations a term has, according to distinct views (direct vs indirect, manual vs all). Direct annotations are annotations made specifically to the term, whereas indirect annotations are annotations made to a parent of the term, and thus inherited by the term. Manual annotations correspond to those made with evidence codes that reflect a manual intervention in the evidence supporting the annotation, while the full set of annotations also includes electronic annotations. Citation features are based on citation of ontology terms based on external resources, in our case PubMed. Finally hybrid features combine some of the previous features into one single value. These features can be mapped onto the change discovery types: structural features belong to their homonymous change discovery type; annotations features can be seen as both data and usage based, since they can be interpreted as both ontology instances and ontology usage; and citation features correspond to the discovery-driven change, since they are derived from external sources. In total we defined 14 features, which we grouped into five sets (see Table 2): *all*, *structure*, *annotations*, *uniformity*, *direct*, *indirect*, 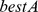 and 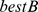. The first three sets are self-explanatory. Uniformity set features were based on [25], where we considered annotations to represent usage. The *direct* set joins direct features of terms, in terms of children and annotations, whereas the *indirect* set joins the same kind of features in their indirect versions. The *best* sets were based on the best features found after running the prediction algorithm for individual features.

**Table 2:** Features and feature sets used for supervised learning.

		all	simple structure	uniformity	annotations	direct	indirect	*best A*	*best B*
Type	Feature	Feature set							
Structural	*dirChildren:* descendants* of a term at a distance of one	+	+			+			
	*all Children:* all descendants* of a term	+	+				+	+	+
	*height:* maximum distance to a descendant	+	+						
	*siblings:* number of terms that share at least one parent*	+	+						
Annotation	*dirManAnnots:* direct annotations given a manual evidence code	+			+	+			
	*dirAnnots:* direct annotations	+			+	+			
	*allManAnnots:* annotations (direct and inherited) given a manual evidence code	+			+		+	+	+
	*allAnnots:* annotations (direct and inherited)	+			+		+	+	+
Citation	*PubMed:* number of articles in PubMed mentioning the term or its children six months before ontology version	+						+	+
Hybrid	*ratioAll:* ratio between *allAnnots* and *allChildren*	+							+
	*ratioDir:* ratio between dirAnnots and dirChildren	+							
	*siblingsUniformity:* ratio between *allAnnots*for the term and the sum of *allAnnots* for its siblings	+		+					
	*parentsUniformity:* ratio between *allAnnots*for the term and the sum of *allAnnots* forits parents	+		+					
	*childrenUniformity:* ratio between *allAnnots* for the term and the sum of *allAnnots* forits children	+		+					

* in the *is_a* and *part_of* hierarchies

Due to the complexity of ontology extension, we have established a framework for the outlining of ontology extension in an applicational scenario. This framework defines the following parameters:

- Extension type:
**refinement**, where a term is considered to be extended if it has novel children terms
**enrichment**, where a term is considered to be extended if it has novel hierarchical relations to existing terms
**extension**, where a term is considered to be extended if it has novel children terms and/or novel hierarchical relations to existing terms
- Extension mode:
**direct**, where a term is considered to be extended if it has new children terms (according to extension type)
**indirect**, where a term is considered to be extended if it has any new descendant terms (according to extension type)
- Term set:
**all** terms
  terms at a given **depth** (maximum distance to root)
  terms at a given distance to **GOSlim** terms
- Time parameters:
**nVer**, the number of versions used to calculate the features

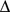
 **FC**, the time interval(in number of ontology versions) between versions used to calculate features and version used to verify extension (i.e. in our dataset, a 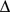
FC of two equals a time interval of one year, since we use ontologies spaced by six months.)

By clearly describing the ontology extension process according to this framework, we are able to accurately circumscribe our ontology extension prediction efforts.

The datasets used for classification were then composed of vectors of attributes followed by a boolean class value, that corresponded to extension in the version to be predicted, according to the used parameters. To compose the datasets we need not only the parameters but also an initial set of ontology versions to be used to calculate features and the ontology version to calculate the extension outcome (i.e. class labels). So given a set of sequential ontology versions 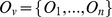, we need to choose one ontology version to predict extension, 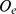, and then based on time parameters 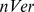 and 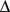
FC, select the set of ontologies to be used to calculate features. For example, for a set of ontologies 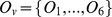, if we chose 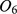 to predict extension, along with 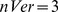 and 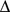
FC = 2, the set of ontologies to calculate features will be 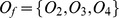.

We tested several supervised learning algorithms, namely Decision Tables, Naive Bayes, SVM, Neural Networks and Bayesian Networks, using their WEKA implementations [36]. For Support Vector Machines, we used the LibSVM implementation with an RBF kernel and optimized the cost and gamma parameters through a coarse grid search. For Neural Networks we used the Multilayer Perceptron implementation, with the number of hidden layers equal to 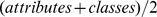, a training time of 500 epochs, and we performed a coarse grid search to optimize the learning rate. Regarding Bayesian Networks, we estimated probabilities directly from the data, and focused on testing different search algorithms, namely Simulated Annealing, K2, and Hill Climbing. Furthermore we had to take into consideration that there are many more terms that are not extended than terms that are, between two sequential ontology versions, which creates unbalanced training sets. To address this issue we used the SMOTE algorithm [37]. SMOTE (synthetic minority over-sampling technique), is a technique that handles unbalanced datasets by over-sampling the minority class and under-sampling the majority class that has been shown to support better classification results for the minority class.

### Evaluation

To evaluate our Ontology Extension Prediction strategy we employed a simple approach: compare our predictions to the actual extension of the Gene Ontology in a future version. To this end we employ another time parameter:

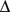
 **TT**, time interval between versions used for training and testing

**Figure 1:**
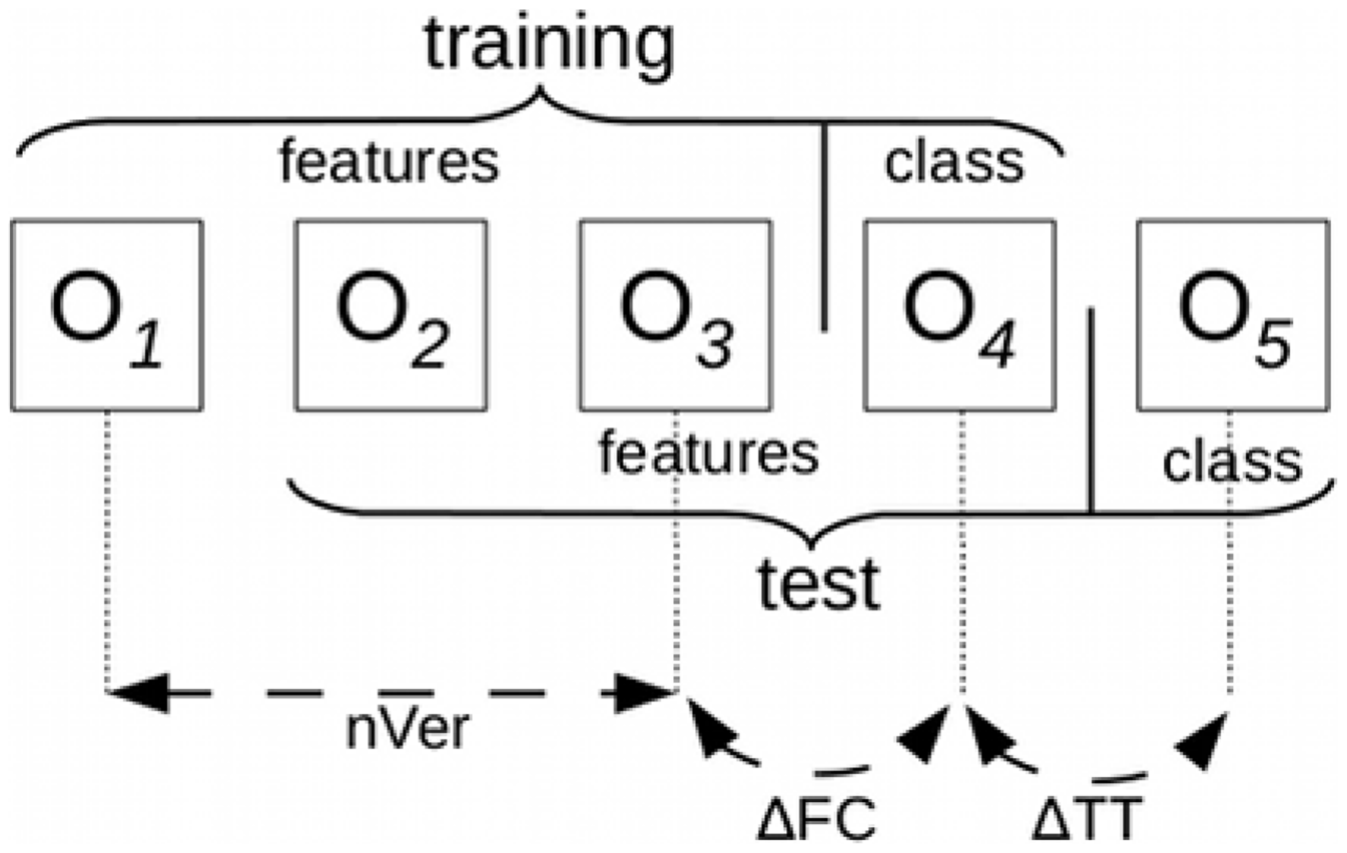
Example of ontology versions to use for training and testing with 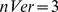
 **,** 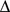
F**C** 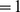 **and** 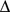 **TT** 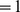 **.**

This time parameter is used to create the test set, by shifting the ontology versions according to 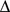
TT. So for instance, given a set of ontologies 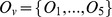 and using 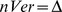
FC = 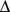
TT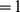, the training and test sets would correspond to the those in Figure 1. Although there may be an overlap in the ontology versions used in a particular training/testing setup, the ontology versions used to determine the class values are always distinct, ensuring that our setup in unbiased.

This approach allows us to compare the set of proposed extensions to real ones that actually took place in a future version of the ontology. We can calculate precision, recall and f-measure metrics, by using the real extension events observed in the more recent ontology version as our test case. These metrics are based on the number of true positives, false positives, true negatives and false negatives. A true positive is an ontology class that our supervised learning strategy identified as a target for extension, and that was indeed extended in the test set, whereas a false positive although having also been identified as a target for extension, was not actually extended. Likewise, a false negative is an ontology class which was not identified as a target for extension, but was in fact extended in reality, whereas a true negative was neither identified as a target nor was it extended in the test set. Precision corresponds to the fraction of classes identified as extension targets that have actually been extended, while recall is the fraction of classes identified as extension targets out of all real extensions. F-measure is a measure of a test's accuracy that considers both precision and recall.
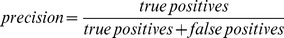
(1)

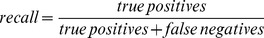
(2)

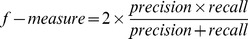
(3)


## Results

When trying to predict ontology extension we are not just focusing on which features are best predictors, but also on how to design the learning process to best support the prediction. Consequently, we are not only trying to find the best prediction set up in terms of features and machine learning algorithms, but also in terms of our strategy's parameters.

### Parameter Optimization

A first step in our experiments was to determine the best term set to use, and to investigate if this was influenced by different parameters. To this end, we tested the following term sets within each GO ontology: all terms, all terms with a depth of 3, 4 and 5, all GO Slim general terms, all GO Slim general leaf terms, all terms at a depth of 1 from the GO Slim general leaf terms, under the same sets of parameters (see Table 3).

**Table 3:** Average term set sizes.

	Average term set size		
Term Sets	Biological Process	Cellular Component	Molecular Function
all	15928	2272.8	8265.6
depth = 3	97.07	21	154
depth = 4	374	112.47	495.33
depth = 5	849	178.47	1093.67
GOSlim	65.27	31.67	-
GOSlim leaves	54.07	26.07	-
GOSlim leaves	1189.93	758.73	-
depth = 1			

To provide a simple basis for our first analysis we focused on the biological process hierarchy and chose a single feature 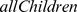 and WEKA's *Decision Table* algorithm with attribute selection using *BestFirst*. Results are presented (unless otherwise specified) using the average f-measure obtained using all possible setups derived from the 15 GO versions available, since we are analyzing a large number of combination of different parameters. So for instance, when using 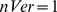, 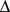
FC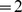 and 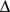
TT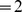, we get a total of ten runs for our prediction evaluation, whereas using 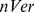 = 3, 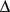
FC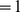 and 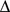
TT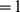 we get only six runs.

Before comparing term sets, we need to analyze the trends between parameter sets. First we focused on extension types and modes (see Table 4). The first clear trend to emerge is that indirect extension is predicted with much more success (0.49–0.86) than direct extension (0.1–0.27). Furthermore, in regards to comparing refinement to enrichment and generic extension, enrichment is poorly predicted, with a performance around 0.20–0.30. The performance for indirect refinement and extension in term sets derived from depth performance is comparable (0.63–0.78), whereas in GO Slim sets refinement is better predicted (0.65–0.86 vs. 0.62–0.65).

**Table 4:** Comparison of extension types and modes.

Term Sets	refinement direct ( 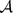 )	refinement indirect ( 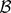 )	enrichment indirect ( 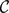 )	extension indirect ( 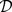 )
all	0.0999 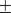 0.07817	0.4919 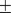 0.03250	0.2009 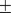 0.09838	0.4674 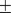 0.03577
depth = 3	0.2704 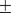 0.22514	0.7896 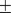 0.05400	0.2955 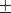 0.22057	0.7495 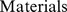 0.05059
depth = 4	0.2176 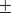 0.17606	0.7083 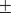 0.03660	0.3429 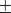 0.17947	0.6790 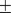 0.04012
depth = 5	0.2313 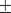 0.14730	0.6348 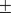 0.04879	0.2898 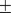 0.14780	0.6268 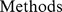 0.05476
GOSlim	0.2024 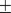 0.22988	0.8637 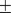 0.05889	0.1722 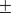 0.21296	0.6530 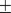 0.30708
GOSlim leaves	0.1635 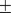 0.21344	0.8553 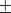 0.06710	0.1003 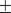 0.17292	0.6470 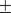 0.30122
GOSlim leaves	0.1523 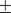 0.13830	0.6529 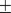 0.06636	0.3168 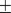 0.10540	0.6243 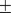 0.07201
depth = 1				

Values are average and standard deviation f-measure for all runs using the 15 ontology versions and a Decision Table algorithm, in the biological process hierarchy. Time parameters: 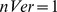, 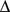
FC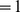, 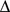
TT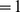.

To clarify this difference, we calculated the average extended proportion for each extension type (see Table 5 for the values for the term set at depth = 4), i.e. the average proportion of extended terms for all GO versions. We verified that the proportion of extended terms is higher for biological process, independently of extension type, followed by cellular component and molecular function, and that the proportion of refined terms is higher than enriched terms, independently of GO term type. This can have an impact on training since there are fewer examples of enrichment.

**Table 5:** Average extended proportion for Gene Ontology according to extension type.

	refinement	enrichment	extension
biological process	0.293	0.103	0.292
cellular component	0.122	0.027	0.124
molecular function	0.076	0.013	0.077

Values are averaged for all GO term at depth = 4 for the 15 ontology versions with an indirect extension mode.

As for the time parameters (see Table 6) and using indirect extension and refinement, the differences are less marked. An increase in the number of versions (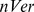) used to calculate the feature values from one to three does not significantly alter the results, and when we extend the interval between versions for feature extraction and extension, we observed an increase in overall performance of about 0.02–0.06.

**Table 6:** Comparison of time parameters.

Term Sets	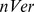 = 1, 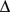 FC 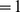 , 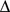 TT 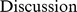 ( 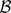 )	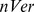 = 1, 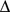 FC 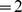 , 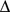 TT 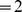 ( 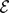 )	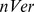 = 3, 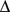 FC 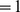 , 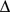 TT 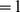 ( 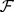 )	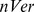 = 3, 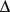 FC 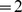 , 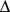 TT 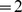 ( 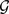 )
all	0.4919 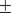 0.03250	0.5301 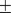 0.01627	0.4890 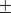 0.03550	0.5301 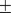 0.01627
depth = 3	0.7896 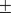 0.05400	0.8177 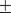 0.05422	0.8152 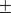 0.04293	0.8005 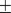 0.07808
depth = 4	0.7083 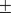 0.03660	0.7520 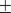 0.03340	0.7267 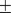 0.04551	0.7437 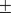 0.04113
depth = 5	0.6348 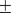 0.04879	0.6962 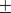 0.04093	0.6526 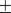 0.04885	0.7101 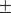 0.03863
GOSlim	0.8637 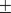 0.05889	0.9020 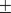 0.07523	0.8264 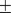 0.06208	0.8869 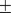 0.08646
GOSlim leaves	0.8553 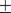 0.06710	0.9004 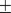 0.06908	0.8378 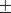 0.05228	0.9046 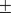 0.07896
GOSlim leaves	0.6529 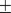 0.06636	0.6748 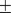 0.07166	0.6624 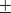 0.06722	0.7021 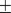 0.04651
depth = 1				

Values are average and standard deviation f-measure for all runs using the 15 ontology versions and a Decision Table algorithm, in the biological process hierarchy. Extension mode: refinement, indirect.

In general, when comparing term sets considering the best sets of parameters (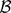, 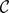 and 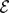, see Tables 4 and 6), it is clear that smaller term sets show a better overall performance. For the remainder of our analysis we will focus on two term sets, *depth = 4* and *GO Slim leaves depth = 1*, which will be referred to as *depth* and *GOSlim* respectively. These sets were chosen to cover both term set strategies and provide a reasonable size set without sacrificing too much performance. We will also from now on focus on refinement and indirect extension, since they represent the primary goal of finding areas of the ontology to extend. Considering time parameters we will use the best overall performers (setup 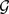: 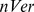 = 3, 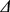
FC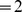, 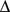
tTT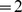).

### Features

The next step in our experiment was to compare different features and feature sets. Table 7 presents the average and standard deviation f-measure values for all features and feature sets using our standard setup.

**Table 7:** Feature and feature sets performance for biological process.

		Term set	
	Features	depth	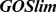
Single	dirChildren	0.6723 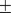 0.02641	0.6662 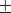 0.04143
	allChildren	0.7437 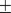 0.04113	0.7021 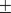 0.04651
	height	0.7426 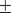 0.03482	0.6854 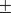 0.04387
	sibsUniformity	0.5814 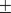 0.15741	0.5283 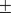 0.15760
	parentsUniformity	0.6336 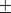 0.03964	0.5430 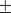 0.17153
	childrenUniformity	0.6469 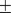 0.05440	0.5899 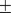 0.08983
	dirAnnots	0.4857 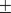 0.15008	0.4964 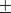 0.06482
	dirManAnnots	0.4838 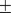 0.10863	0.4748 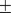 0.05278
	allAnnots	0.7335 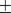 0.03663	0.6821 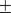 0.03579
	allManAnnots	0.7452 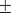 0.02882	0.6965 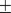 0.04940
	PubMed	0.5960 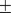 0.03933	0.6552 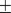 0.04709
	ratioAll	0.6850 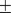 0.04231	0.6192 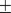 0.03266
	ratioDir	0.5735 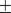 0.11476	0.5856 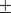 0.03939
Sets	all	0.7459 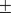 0.03675	0.7801 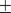 0.0525
	structure	0.7431 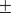 0.02543	0.6906 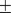 0.04546
	uniformity	0.6523 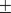 0.06109	0.5727 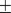 0.19389
	annotations	0.7396 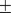 0.02893	0.6949 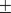 0.04771
	direct	0.6661 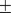 0.03684	0.6569 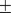 0.05436
	indirect	0.7641 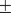 0.03242	0.6883 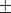 0.06412
	bestA	0.7415 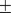 0.04270	0.7704 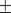 0.04450
	bestB	0.7550 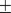 0.03049	0.7750 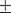 0.04265

Values are average and standard deviation f-measure for all runs using the 15 ontology versions and a Decision Table algorithm. Time parameters: 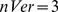, 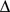
FC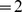, 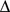
TT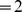.

When using single features, the best performers are 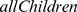, 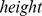 and 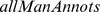, with average f-measure values around 0.74 in the 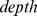 set and 0.69 in the 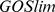 set. When using sets of features, in the 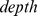 set the top performers are *indirect*, 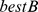 and 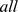, with values between 0.75 and 0.76, whereas in 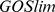 they are 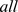, 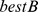 and 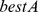, with values between 0.77 and 0.78. Using feature sets insetad of single features has a positive impact on performance in the 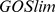 set, which is not noticeable in the 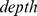 set.

### Gene Ontologies

So far we have focused on predicting refinement within the biological process ontology. Tables 8 and 9 summarize the results obtained for the molecular function and cellular component hierarchies, showing the top three features and feature sets for each term set. For molecular function we show only results for the term set based on 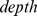 since there is no 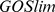 subset.

**Table 8:** Summary of feature and feature sets performance for cellular component.

		Term set	
	Features	depth	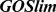
Single	allManAnnots	0.7085 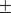 0.07487	0.6068 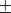 0.06908
	allChildren	0.6800 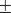 0.11041	0.5650 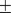 0.09469
	ratioAll	0.6604 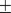 0.04485	0.4636 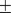 0.02932
	height	0.6450 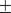 0.08744	0.5248 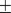 0.08186
Sets	bestB	0.7210 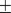 0.08485	0.5174 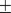 0.08370
	bestA	0.7155 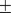 0.09198	0.4758 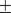 0.11213
	annotations	0.7046 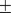 0.08523	0.6198 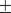 0.03661
	all	0.6916 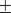 0.11118	0.4367 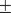 0.14839
	structure	0.6890 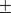 0.13975	0.5985 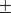 0.04716

Values are average and standard deviation f-measure for all runs using the 15 ontology versions and a Decision Table algorithm. Time parameters: 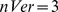, 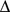
FC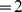, 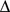
TT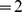.

**Table 9:** Summary of feature and feature sets performance for molecular function.

		Term set
	Features	depth
Single	allChildren	0.6650 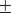 0.07957
	allManAnnots	0.5898 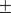 0.07267
	height	0.5633 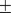 0.08577
	dirChildren	0.5577 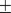 0.06710
	allAnnots	0.5572 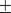 0.08084
Sets	bestA	0.6441 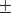 0.04625
	indirect	0.6395 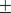 0.07485
	bestB	0.6285 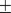 0.06971
	all	0.6218 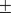 0.04873
	structure	0.6168 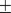 0.06450

Values are average and standard deviation f-measure for all runs using the 15 ontology versions and a Decision Table algorithm. Time parameters: 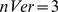, 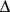
FC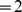, 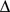
TT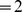.

Although average f-measure is generally lower for both molecular function and cellular component, than for biological process, 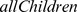 and 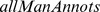 continue to be among the best features. Furthermore, for cellular component the 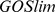 set shows a worse overall performance than the 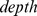 set, in disagreement with what happens in biological process.

### Supervised Learning Algorithms

**Figure 2:**
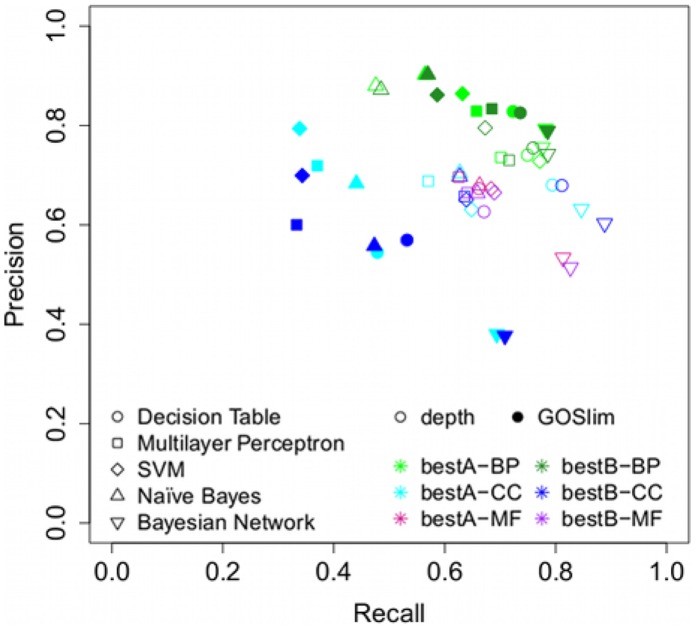
Average precision and recall for several supervised learning algorithms using the 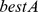
 **and** 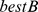 **feature sets, depth and GO Slim based term sets and** 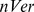 **= 3,** 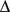 **FC** 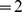 **,** 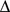 **TT** 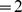 **in all three GO hierarchies.**

In addition to Decision Tables, chosen due to their simplicity, we also tested several other commonly used supervised learning algorithms, namely Naive Bayes, SVM, Neural Networks (Multilayer Perceptron) and Bayesian Networks, using their WEKA implementations. Figure 2 shows a plot for precision and recall for the best feature sets using these algorithms.

When applying different learning algorithms, we still see that overall biological process has the best performance, followed by molecular function and cellular component. Likewise, the general performance in the 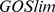 term set is better than the one in the depth term set for biological process, whereas it is the reverse for cellular component.

Looking in with more detail at the biological process results, the difference between feature sets is small, so we will not distinguish between them in our analysis. Naive Bayes gives the top precision values (0.87–0.90) but the lowest recall (0.48–0.57), whereas Bayesian Networks have the highest recall (0.78–0.79) with precision values between 0.74 and 0.79, which correspond to average f-measures between 0.76 and 0.79. SVM, Decision Tables and Multilayer Perceptron have performances in between these with both recall and precision values clustered around 0.70.

In molecular function, the highest precision is given by Multilayer Perceptron at 0.70 for 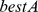, and Multilayer Perceptron, SVM and Naive Bayes for 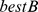 at 0.66–0.67. The highest recall is found in 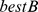 by Bayesian Networks at 0.83. Best average f-measure is achieved by SVM at 0.66 for both 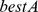 and 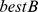.

In cellular component, there is a marked difference between the performance in the depth term set and in the 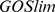 set, with the latter having in general a much lower recall, around 0.40, except when using Bayesian Networks, where recall rises to around 0.7, but at the cost of precision. There is also a visible difference between term sets, with 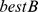 having in general a lower precision for the 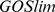 set, which is not apparent in the depth term set. In the depth term set the best performing algorithms are Decision Tables and Bayesian Networks, with recall around 0.8 and precision above 0.6. Decision Tables achieves the top performance with an average f-measure of 0.72 for 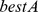.

### Comparative Evaluation

**Figure 3:**
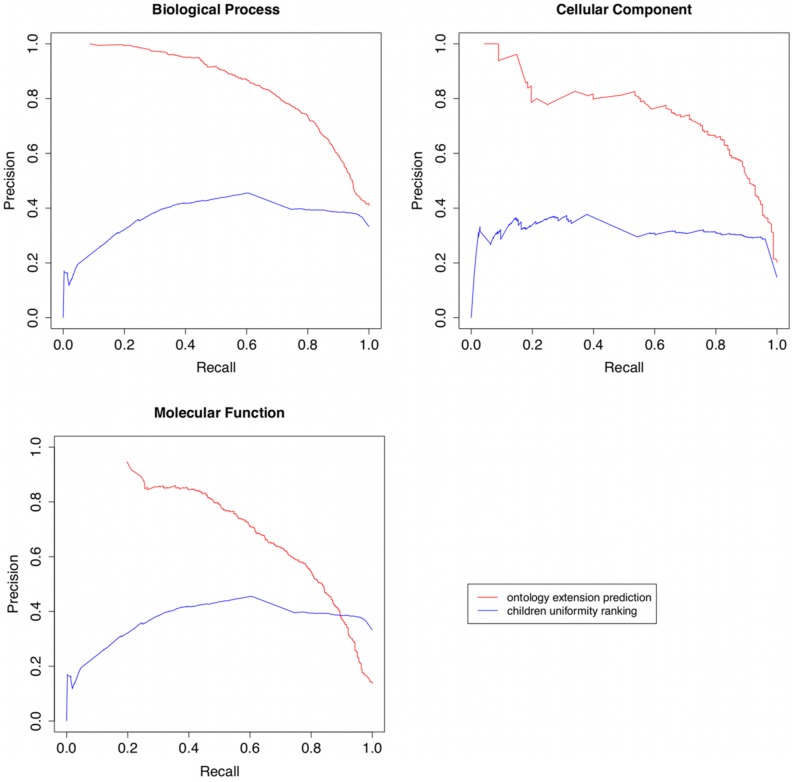
Precision/Recall plots for refinement prediction based on Stojanovic's children uniformity and our own strategy.

To provide a basis for comparison, we implemented Stojanovic's browsing uniformity measures [25] and evaluated them on predicting ontology evolution for GO. For link usage we used annotation frequency. Since this strategy does not identify targets for extension, but rather ranks classes according to their uniformity, we evaluated this strategy plotting precision-recall curves for all ontology versions used. Figure 3 shows precision/recall plots for children uniformity, using one version of the ontology to calculate uniformity and predicting refinement for a following version in our dataset, alongside the plots for our prediction strategy best configuration (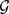, 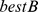). For both cases we used the term set based on a depth of 4 and a distance between training and testing of two versions.

For plotting our strategy instead of relying on the binary labels output by the classifier, we used the probabilities for each instance to be true (i.e. refined), so that the generated plots are more directly comparable to those produced by the uniformity strategy, allowing a more granular calculation of precision at different recalls to allow for a threshold based evaluation. Consequently, the presentation of the results of our strategy in these plots differs from the presentation in previous tables.

The prediction results for all ontologies were combined together in the plotting of the Precision/Recall plots to provide a better visualization of results. As it is patent in the plots, our strategy has a considerably improved performance in all three GO ontologies, with curves closer to the top right corner, which are indicative of both higher precision and recall. The uniformity strategy performed worse in all cases, except at higher recall values in molecular function.

The other uniformity strategies (parents and siblings) have an even lower performance than that of children uniformity.

## Discussion

Change capturing through prediction of ontology extension is a complex issue, due to the inherently complex nature of ontology extension itself. Ontology extension can be motivated by implicit or explicit requirements, which have very different mechanisms. Implicit requirements are in principle easier to predict since they do not change between ontology versions, whereas explicit requirements, which are created by experts to adapt the ontology to a novel conceptualization or change in the domain, are much harder to predict. Our strategy, by virtue of being based on learning using past extension events, cannot distinguish between these two types, and thus attempts to predict extension regardless of it being motivated by implicit or explicit requirements. To capture both kinds of requirements we use a set of ontology based features that not only contemplate intrinsic features, such as structural ones, but also extrinsic ones, such as annotations and citations.

The assumption that extension can be predicted based on existing knowledge, either in the form of the ontology itself or its usage, is acceptable regarding the more common extension events, but is not applicable to extension events that are the result of deep restructuring or revision of existing knowledge. These extension events are part of a complex ontology change that also includes deletions and modifications. As such, these more complex changes are not the object of our strategy. In fact, one of our strategy's goals is to speed up the process of accomplishing the simpler extensions, to give experts more time and resources to focus on the more complex events.

One very relevant aspect of our evaluation strategy is that we compare our results to the real extension events that occurred in more recent versions of the ontology. This means that although some of our predictions are conceptually correct, they may not have yet been included in the ontology version used for testing and will thus be considered incorrect. This will have an impact on precision values, since we might be capturing needed but still unperformed extensions, and then be considering them to be incorrect in our evaluation. Due to this line of thought, we might then give preference to strategies that increase recall even if at the cost of precision. However, this could have the negative effect of including many incorrect predictions in our output, which is not desirable in a semi-automated ontology extension system. As such we have chosen to base our evaluation on f-measure, to provide balanced precision and recall.

A basic requirement of our strategy is to be able to access several versions of the ontology to consider. The minimum set of ontology versions it requires is two: one which will be used to calculate the features, and a second one, more recent than the first, from which we will extract the class labels to train the model. It then becomes crucial to define the interval between the versions to use. In our test case using the Gene Ontology we decided on versions with an interval of at least 6 months, based on the intuition that a smaller interval would not provide us with sufficient extension examples to be able to train a model. This intuition was shown to be a good approximation, since as seen in Text S1 and Table S1 in Text S1, when using monthly versions we do in fact have a very low number of positive examples.

### Parameters

Due to the complexity of ontology extension, particularly in such a large ontology as the Gene Ontology, our prediction strategy has to account for several parameters that help circumscribe our effort. One such parameter, extension type, was designed to capture the different types of extension: refinement and enrichment. We have found that refinement is considerably easier to predict than enrichment, with refinement having a greater average f-measure by between 0.3 and 0.7. There are two likely explanations for this difference: on one hand, there are many more refinement events between ontology versions than there are enrichment events (see Table 5), which will provide a better support for supervised learning; on the other, the features used may be better correlated to refinement than to enrichment.

Another parameter related to extension, is its mode, direct or indirect. Predicting direct extension, i.e. exactly which terms will be extended in a future version, should be the ultimate goal of an ontology extension prediction strategy. However this was proven to be a difficult task, which is unsurprising given the multitude of different processes that can lead to extension, and also the fact that on average new terms correspond to about 5% of all terms in an ontology version (see Table 1). This follows the trend found in our previous work [24], where we analyzed the extension of GO and found that insertions of new terms often occur together.

To address this issue we focused our prediction efforts in slices of the ontology, and defined the extension that happens within the subgraphs rooted in terms within these slices as indirect extension. Focusing only on the term sets thus defined greatly improved the performance of our strategy (Table 4), with average f-measures for the prediction of refinement of biological process increasing from 0.49 to 0.65–0.86 depending on the term set considered.

Predicting for a subset of the ontology is supported by our previous finding [24] that extension frequently happens by branches and that introducing terms closer to the root has a large impact on the overall structure of the ontology. Consequently, determining which term sets to use must be a compromise between enough specificity to be useful, but enough generality to provide a good enough balance of positive and negative examples. We determined six such subsets, following two distinct approaches: based on distance to root and based on GO Slim general.

We chose distance to root for its simplicity in creating a middle layer of GO terms. However, since terms at the same distance to the root do not always have the same degree of specificity, we also used GO Slim general as a basis for our other strategy. By using GO Slim general we were attempting to capture a similar degree of specificity among terms, specific enough to provide a useful prediction and general enough to allow for branch extension prediction. We tested three different sets within each approach, each yielding different term set sizes. Since molecular function does not have a GO Slim general, we only tested distance to root (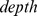) based sets.

For both approaches, the smaller the data set the better the results. This can in did be due to the fact that in smaller data sets there is a better balance of positive and negative instances, which despite our use of SMOTE to balance the training sets, still has an impact on training the models. However, we are not interested in very small term sets, since they would not provide enough specificity to change capturing for ontology extension. Considering this we focused on the term set defined by terms at a distance of one from GO Slim leaf terms, which corresponds to an average term set size of 1189 for biological process and 758 for cellular component, and on the term set defined by terms at a distance of four to the root, which corresponds to sizes around 370, 460 and 100, for biological process, molecular function and cellular component respectively.

The final parameters in our strategy are those related with time: 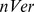, 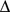
FC and 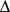
TT. We found that the influence of the number of versions used to derive the features was minimal. Regarding the intervals between versions for feature and class, and for training and testing, we found that increasing those intervals from six months to one year resulted in an increase in performance (about 0.03 to 0.06), which is likely due to the fact that the number of positive examples is larger when considering a larger interval between versions. Considering these results, we focused on the setup of 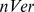 = 3, 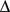
FC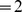 and 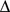
TT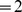.

### Features

Although the parameters previously discussed represent the basis of our strategy, by defining exactly on what the prediction is focusing, it is the features used to support prediction that are essential to be able to capture extension events. Using the best parameter setup we investigated a set of thirteen single features, also arranged into eight sets, and found some interesting trends. In the 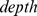 term set, the single features 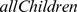 and 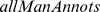 were among the top performers for the three GO hierarchies. But in the 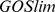 for biological process feature sets performed better than single features, whereas in cellular component this difference was not apparent. However, the feature sets composed of the best single features (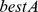 and 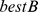) were shown to provide the better performances across the board, with the exception of the 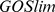 set in cellular component. It is interesting to note that although using just structural or just annotation based features can provide in most cases a performance comparable to combining them, which can simplify our strategy, using a combination of the best single features can in some cases improve performance.

One of the most obvious patterns we get from these results is that terms with a lot of children terms or a lot of total annotations tend to be extended. It is arguable that for larger subgraphs, the probability of an extension event occurring is greater, given that there are more terms in it. However, to support the theory that the only factor involved is indeed the number of terms in the subgraph (i.e allChildren), we would have to consider that the probability of extension for any given term is equal. Intuitively, this does not appear to be a valid assumption, since it would mean that the extension of GO does not follow any particular direction. Nevertheless, we investigated this possibility by comparing the distribution of real refinement events for 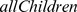 intervals, with the probability density function of a binomial distribution for at least one success for the same 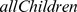 intervals. Figure S3 shows that the two distributions are significantly different, thus supporting the notion that although the number of children has an influence in the refinement probability, the probability of refinement is not the same for all terms. From these results we can hypothesize that the number of children a term has is related to its probability of refinement, because it reflects an increased interest in that area of the ontology.

Furthermore, the total number of annotations is influenced by the total number of children, since the annotations of the children contribute to the total number of annotations of the parent. To take this into account, we created the feature ratioAll to mitigate the influence of the number of children on the annotation data. Although this resulted in a decrease in f-measure of around 6%, compared to either feature separately, it is still a better performance than most other features. This gives further support to the notion that areas which attract a larger interest (in this case patent in the number of annotations) become the object of more refinement events.

Although these simple notions appear quite intuitive, and we could in principle derive a simple generic rule based on the number of children, in order to support automated change capturing, we need to establish the best separation possible between targets and non-targets for refinement, which is best achieved by employing supervised learning.

### Supervised Learning

The results discussed so far were all based in Decision Tables, a simple supervised learning algorithm. We also tested other algorithms, but realized that although other algorithms such as SVM, Neural Networks and Bayesian Networks were capable of providing a better performance, and specifically in the case of SVM and Neural Networks of being parametrized to privilege either precision or recall, Decision Tables was still able to provide generally good results comparatively, without requiring parameter optimization.

We were particularly interested in the performance of Bayesian Networks, since our attributes are not independent, but in fact are temporally related when we consider multiple ontology version for feature extraction. For instance the value of 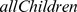 in one version depends on its value in the previous one. However, we did not find a marked difference between Bayesian Networks and other approaches, so this dependency appears to not be very relevant for our current strategy.

Another particularly interesting aspect is that most machine learning algorithms, including the ones that were used, assume that instances are all independent and identically distributed. However, the dataset instances correspond to GO terms which are hierarchically related through the GO structure. Although the inclusion of features that describe the neighboring area tried to capture this aspect (e.g. siblings, and all the uniformity features), we still believe it was not properly contemplated by the proposed setup. The hierarchical relations between instances may be affecting the experiments considering the full set of terms, since they are not being captured by the representation. In the subset of terms dataset, their influence would not be as strong, since there are fewer hierarchical relations between instances.

### Comparative Evaluation

To complete our evaluation, we compared our strategy to the one proposed by Stojanovic *et al.* [28] based on uniformity. In general, the uniformity based strategy performed worse than our own. This however is a consequence of Stojanovics approach having been designed to support the manual extension of an ontology that adapts to user's needs, whereas in our setting we have an ontology that models knowledge about a domain, whose extension is caused by many different aspects. Curiously, when transforming the uniformity metrics into features for classification, we achieve a better performance (Table 7) than when using them as intended by the authors, as a simple criteria for ranking.

### Applying Extension Prediction

The output of our extension prediction methodology is a list of ontology classes, which are the roots of subgraphs that correspond to ontology areas which have been predicted as good candidates for extension. Our methodology is applicable to the most simple yet most frequent type of ontology change, the addition of new elements. It is not suited to predict more complex changes such as a reorganization of an entire branch of the ontology. As such, the ontology extension prediction can be used to speed up the process of extension in these simpler cases, by allowing ontology developers and/or ontology learning systems to focus on smaller areas of the domain. This frees the experts to spend more time focusing on the more complex changes that cannot be predicted.

Automated ontology learning systems can also use the list to focus their efforts on the identified areas. For instance, most ontology learning systems employ a corpus of scientific texts as input, and their performance is tightly coupled to the quality of such corpora. If our candidate list is used to guide the creation of specific corpora for the areas to extend, it can have a positive impact on the performance of such strategies.

**Figure 4:**
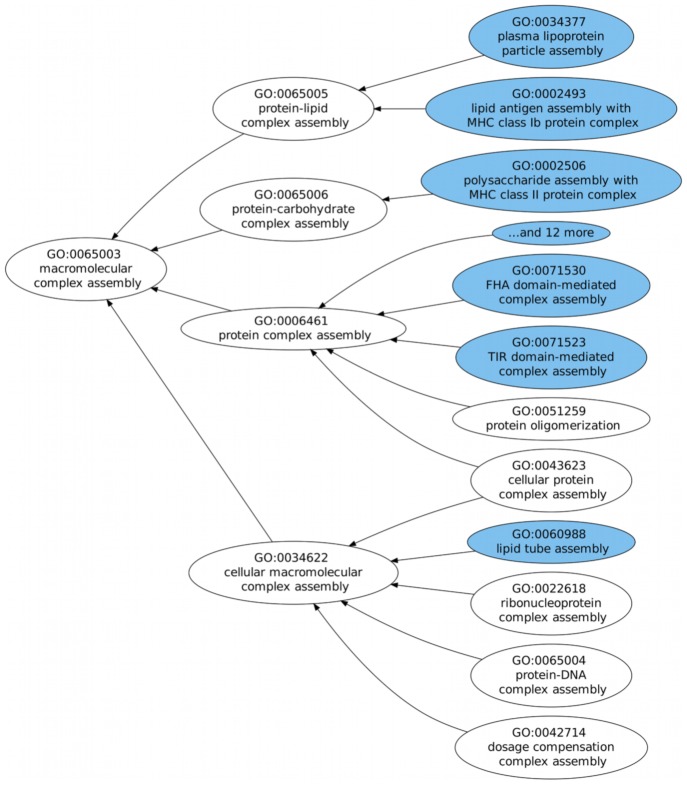
Example of predicted extension in the Molecular Function hierarchy. Extension was predicted for the root term and occurred at a distance of two edges, in every subclass.

**Figure 5:**
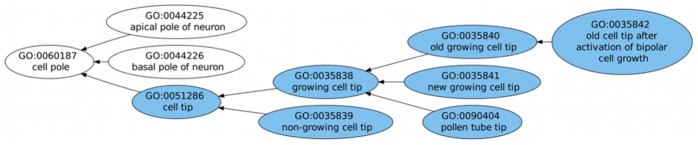
Example of predicted extension in the Cellular Component hierarchy. Extension was predicted for the root term and occurred at a distance of one edge, with the addition of a whole new branch.

**Figure 6:**
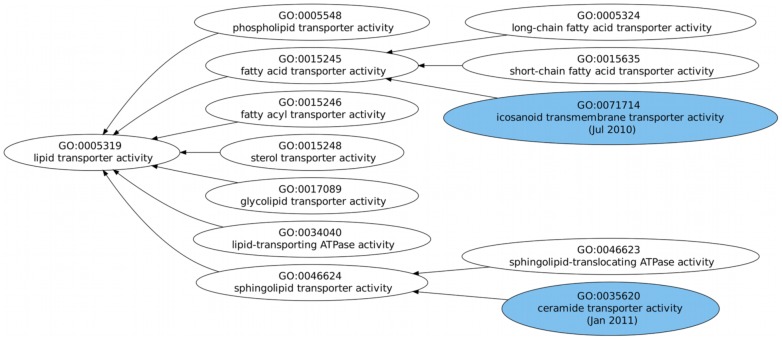
Example of predicted extension in the Biological Process hierarchy. Extension was predicted for the root term and although it did not occur in the version for which it was predicted (January 2010), it did in fact occur in later versions, with the addition of one new sub-subclass in July 2010 and another in January 2011.

We have chosen to highlight three examples of the results given by our ontology extension prediction system, two successful ones (Figure 4 and Figure 5), where the predicted areas were in fact extended in the version for which extension was predicted, and one indirectly successful one (Figure 6), where although the extension did not occur when predicted, it did in fact happen at later versions of the ontology.

In Figure 4, extension was predicted for the subgraph rooted in “macromolecular complex assembly". Since we are predicting indirect extension, the addition of new subclasses can occur at any point in the subgraph. In this case, the GO term has four direct subclasses, and all of them gained new subclasses in the future version for which we were predicting. In Figure 5, extension was predicted for the area of “cell pole". In the version used to train the model, “cell pole" had two subclasses “apical pole of neuron" and “basal pole of neuron" but in the version for which extension was predicted, “cell pole" gained a whole new branch rooted on a new subclass for “cell tip". These two examples showcase two different extension patterns: in the first, extension occurs throughout the subgraph, whereas in the second it corresponds to the addition of a single but large branch.

In Figure 6 extension was predicted in the subgraph of “lipid transporter activity" for the version of January 2010, but no extension took place. However in later versions of July 2010 and January 2011, extension did occur by the addition of two new sub-subclasses. This is an example of how our evaluation strategy may be too stringent when considering these cases false positives, since they can eventually undergo extension at later versions.

### Summary and Future Work

Ontologies are crucial to handle the challenges of an increasingly data-driven world. However, ontologies themselves face this challenge, since the effort to keep them updated in face of the new knowledge that is produced on a daily-basis is never complete. To support this effort, some of the processes involved in ontology evolution can be automated, in order to reduce the time and resource investment made by expert curators.

In this work we present such a strategy for the first step of ontology evolution: change capturing. Our strategy is based on predicting areas of the ontology that will undergo extension in a future version, by applying supervised learning over features of previous ontology versions. We applied our strategy to the Gene Ontology, where we obtained encouraging results with average f-measure reaching 0.79 for prediction of refinement for a subset of relevant biological process GO terms.

In addition we defined a framework to better define extension in an applicational context, that can be applied to ontologies with versioning, as is the case of OBO ontologies and many of its candidates. This framework is crucial to provide a better understanding of the various nuances of ontology extension, and as such support ontology extension prediction efforts.

We find that two particular characteristics of our strategy can be improved, namely the selection of ontology versions to use and the selection of the term set. Both of these can benefit from recent works on ontology evolution [31], [38] from which we can gather useful information to guide the selection process. For the ontology versions, as we have discussed above, there is a need for a minimum of changes between versions to allow for the training, and by using these works we can pinpoint ontology versions that have enough changes between them. In what concerns the term set, we can benefit from the identification of stable and evolving regions of the ontology, and thus dynamically define distance to root based on this criteria, i.e. for stable regions we predict for terms further away from the root, whereas for evolving regions we stay closer to the root.

Although we applied our strategy to the Gene Ontology, it is applicable to any ontology with multiple versions available, which is becoming increasingly prevalent, as ontologies in biomedicine mature. The performance of our strategy on other ontologies is still to be tested and the next logical testing ground for the proposed methodology are smaller ontologies which lack the maturity and funding of larger ontologies such as GO. Several ontologies would be interesting to explore, such as the Pathway Ontology or the Ontology of Physics for Biology, which provide several versions but are much more recent and quite smaller than GO. The success of our strategy on GO using simple structural data is encouraging, since most ontologies lack such a rich annotation corpus as GO's, but all provide structural data which can be explored.

Predicting the extension of an ontology can have a positive impact in ontology evolution processes, be they manual or automated, by focusing efforts and reducing the amount of new information that needs to be processed. Moreover, OBO's principles of maintenance and orthogonality strongly advocate for the existence of a single ontology for each domain that is progressively enhanced, rather than a myriad of niche ontologies. Consequently, strategies that aid in the evolution of existing ontologies, as the one proposed here, present themselves as relevant contributions to the end goal of ontologies in biomedicine.

## Supporting Information

Figure S1F-measure for refinement prediction for separate ontology versions using Decision Tables with the 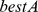 feature set and 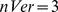, 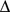
FC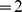, 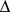
TT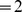.(EPS)

Figure S2Percentage of positive examples for training models for refinement prediction for separate ontology versions using Decision Tables with the 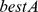 feature set and 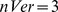, 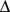
FC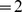, 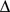
TT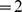.(EPS)

Figure S3Relation between number of 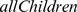 and refinement probability. The label ‘observed’ corresponds to the real observed refinement events, whereas ‘expected’ to the refinement proportion expectable following the binomial distribution. Presented values correspond to the GO version of June 2010, but other versions present a very similar behavior. 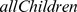 and refinement values are averaged within intervals of size 10. These intervals were calculated by ordering the terms according to the their 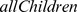 number in ascending order, and then generating equal sized intervals.(EPS)

Text S1 In the supplemental text we present two additional studies: one on using consecutive monthly versions of the ontologies instead of six-month separated ones, and another on the evolution of prediction, to investigate whether prediction performance is comparable through time.
(PDF)
