# Supplementary material for: Predicting the Extension of Biomedical Ontologies
Source: PLoS Comput Biol. 2012 Sep 13;8(9):e1002630. doi: 10.1371/journal.pcbi.1002630 (PMC3441454; doi:10.1371/journal.pcbi.1002630)
Supplement: Text S1 — In the supplemental text we present two additional studies: one on using consecutive monthly versions of the ontologies instead of six-month separated ones, and another on the evolution of prediction, to investigate whether prediction performance is comparable through time. (PDF) [file pcbi.1002630.s004.pdf]

Predicting the Extension of Biomedical Ontologies

Catia Pesquita*, Francisco M. Couto
Faculty of Sciences, University of Lisboa, Portugal
∗ E-mail: cpesquita@xldb.di.fc.ul.pt

1 Supplemental Information

1.1 Consecutive version prediction

Although we decided on using six months as the minimum interval between consecutive ontology version, we also did a small study on using monthly versions. As a first step we investigated the average number of refinements made in consecutive monthly versions between May 2010 and October 2010 (six versions), within each GO ontology (Table S1).

Table S1. Average number of refined and non-refined GO terms

GO ontology	refined terms	non-refined terms
depth=4	consecutive versions	
biological process	84.00 ± 22.967	594.5 ± 18.554
molecular function	9.50 ± 9.604	449.25 ± 259.479
cellular component	3.25 ± 3.418	114.5 ± 66.130
GOSlim leafs depth=1		consecutive versions
biological process	118.25 ± 41.583	1156.75 ± 41.583
cellular component	6.5 ± 7.762	604.0 ± 348.785
depth=4	six month separated versions	
biological process	105.89 ± 22.605	224.22 ± 36.0917
molecular function	41.89 ± 16.010	466.89 ± 20.572
cellular component	11.78 ± 2.779	92.56 ± 44.919

The number of refined terms between consecutive versions is much lower than using a six month interval, as expected. In the molecular function and cellular component ontologies, this has a great impact on prediction, since between some versions there were actually no refinements at all. This of course precludes prediction for these cases, but even when there are refinements, the number of positive training examples is still much lower than the negatives, making it either impossible to train a model, or making the model over fitted, and hence have a lower performance on the prediction task.

1.2 Evolution of prediction

All presented results have been averages for all predictions made using a given setup. We were however also interested in verifying if there is any trend in extension prediction, so we plotted individual f-measure values for all three GO ontologies using our standard setup (Decision Tables, $bestA$, $nVer = 3$, ΔFC= 2, ΔTT= 2, refinement, indirect). Figure S1 shows this plot, where we can observe that for biological process, there is very little variation across time, whereas for molecular function and cellular component there is greater variation.
We hypothesized that this could be due to variations in the number of positive examples between different versions, which could be impacting the training of the model. To investigate this we calculated the percentage of positive examples within each dataset, and plotted this in Figure S2.
